# Comparison of Conventional and Microwave Assisted Heating on Carbohydrate Content, Antioxidant Capacity and Postprandial Glycemic Response in Oat Meals

**DOI:** 10.3390/nu10020207

**Published:** 2018-02-14

**Authors:** Joanna Harasym, Remigiusz Olędzki

**Affiliations:** 1 Bio-Ref Lab, Department of Biotechnology and Food Analysis, Faculty of Engineering and Economic, Wrocław University of Economics, 53345 Wroclaw, Poland; 2 Department of Biotechnology and Food Analysis, Faculty of Engineering and Economic, Wroclaw University of Economics, 53345 Wrocław, Poland; remigiusz.oledzki@ue.wroc.pl

**Keywords:** microwave assisted heating, oat flakes, oat bran, antioxidant capacity, carbohydrate polymers, β-glucan, glucose, glycemic response

## Abstract

Minimally processed cereal breakfast products from whole grain entered the market due to consumer demand of more nutritional food with more controlled sugar release. However, the subsequent processing of such products with different cooking methods in the consumer’s kitchen may lead to significant differentiation of their nutritional value. Therefore, the evaluation of the impact of frequently used cooking methods on a final quality of breakfast cereals meal is needed. The present study investigates how the two different methods of heating, conventional and microwave (MW) assisted, affect the carbohydrate content, profile and resulting glycemic index of so prepared food as well as the antioxidant activity of meals. Two products available on the market—oat bran and flakes—were used. The highest starch content in fluid phase of oatmeal was detected in samples heated for 3 min with microwaves, regardless the type. The lowest starch content was obtained for 5 min MW heated flakes sample. The total content of glucose was about 1.5 times lower in bran vs. flakes oatmeal. The highest β-glucan content in fluid fraction was also observed for bran meal but its release was independent of applied conditions.

## 1. Introduction

After years of white flour domination on the cereal food products market, the renaissance of whole grain is very noticeable nowadays [1] and its pro-healthy activity is quite well located in the consumer awareness. It was assessed by an extended study of cereal grain bioactive components, mainly from its external aleuronic/bran fractions and embryo [2]. However, despite all the health benefits of whole grain consumption, eating it in raw form is not recommended; therefore, such products need to be processed. The milling industry partly transforms the whole grain and offers on the market the dehulled whole grain, puffed whole grain, flakes, cut grain or bran. The subsequent processing stage is transferred to customers, who can face severe problems when preparing dishes which are either nutritionally equilibrated or not overloaded with easily accessible carbohydrates.

The whole grain cereal of high nutritional quality is oat, being the abundant source of dietary fiber, especially the soluble one, as well as antioxidant compounds and slowly digestible starch [3]. Several national and international food safety agencies have issued their authorization for using the so-called “health claims” on products containing specified amounts of oat compounds, either chemical (1–3, 1–4-β-d-glucan) or nutritional (oat fiber) [4,5,6]. Recent studies have revealed the other mechanisms of its bioactivity beyond the physical mechanics of the viscous film in the gastrointestinal tract, being intensive metabolic interaction with intestine, stomach, spleen and liver tissues [7,8,9]. Because several studies reported that different processing of oat impacts nutrients content [10], especially the availability of antioxidants and 1–3, 1–4-β-d-glucan, a detailed study is needed to find the best and nutritionally equilibrated method of preparing food from oat. The main contributors to an overall antioxidant activity of oat fractions are still under study. The antioxidant activity has been assigned to trace elements and minerals, vitamins, carotenoids, polyphenolic acids, alkylresorcinols, betaine, choline, sulfur amino acids, phytic acids, lignans and avenanthramides (mainly located in the bran and germ fractions) [11]. The avenantramides (the conjugate of one of three phenylpropanoids (*p*-coumaric, ferulic, or caffeic acid) and anthranilic acid (or a hydroxylated and/or methoxylated derivate of anthranilic acid) are assumed to be main sources of antioxidant activity of oat (donating a hydrogen atom to a radical) [12]. However, pure β-glucan has also shown antioxidant activity both in vitro [13] and in vivo [14]; therefore, it is hard to find the main compound responsible for antioxidant activity in multi-component extracts. Additionally, because oat products are not eaten raw, the thermolability of active molecules should be taken into account. On the other hand, thermal processing also liberates other compounds from plant matrix, which can further contribute to the total antioxidant activity of oatmeal.

The meta-analysis of mixed oat products including whole oats and extracted oat 1–3, 1–4-β-d-glucan revealed a significant change in blood fasting glucose with high factor of heterogeneity (*I*^2^ = 97%) [15]. Additionally, other studies revealed that oat product may be beneficial to healthy subjects, hypercholesterolemic and diabetes type 2 patients [16] which amplified massive information action about healthy impact of oat consumption. The minimally processed oat whole grain products available on the market are breakfast steel cut oats or rolled oats (oat flakes) of different level of pre-gelatinization and native grain shape destruction as well as oat bran. The preparation procedure of typical breakfast from whole grain products involves heating or cooking stage. The time of cooking depends on previous industrial treatment of cereal products, which means that those minimally pre-processed need longer cooking to obtain nutritionally equilibrated meal. The industrial processing significantly changes nutritional compounds release and their mutual interactions in prepared food [17,18]. Therefore, it can be supposed that different whole grain products cannot be prepared with the same cooking method to obtain similar nutritional value of a meal.

The following study compares two types of oat breakfast products in relation to nutrients extractability into water during different cooking methods, i.e., short- and longtime microwave and conventional heating. The two applied heating methods differ significantly one from another. The conventional cooking is based on conduction and convectional current transporting heat from external sources through the whole volume of food, while in-situ heat generation, resulting from interaction with microwaves, not only removes the convectional transfer restriction but also creates the superheated micro-sites facilitating the liberation of active compounds. Microwave heating was successfully used as extraction support [19], for inactivation of undesired enzymatic activity such as β-glucanolytic [20] or lipolytic [21], and for changing the functional properties of gluten-free flours [22,23]. The microwave frequency typical for kitchen ovens in Europe is 2450 MHz. Some research on effects of temperature dependent properties in electromagnetic heating was done on salmon fillets providing overall conclusions that, for frequencies above 2000 MHz, both constant and variable (temperature dependent) dielectric properties have identical temperature profile, while the volumetric power absorption increases or remains the same for the microwave heating [24].

Therefore, the evaluation of microwave heating application to assess the nutrients release profile of oat breakfast products seems to be needed. To the best knowledge of the authors, this is the first study comparing those two different preparation methods of whole grain breakfast products aiming at assessing carbohydrates content, their interaction during extraction, antioxidant activity of extracts and final impact on glycemic response.

## 2. Materials and Methods

### 2.1. Materials and Chemicals

Two different whole grain cereal breakfast products from oat *(Avena sativa* L.) were purchased on local market: flakes (F) (Melvit, Poland) and bran (B) (Sante, Poland). For antioxidant capacity assessment, 2,2-diphenyl-1-picrylhydrazyl (DPPH), 2,2′-azino-bis (3-ethylbenzothiazoline-6-sulfonic acid), Folin–Ciocalteau’s reagent, gallic acid, sodium carbonate, and Trolox (6-hydroxy-2,5,7,8-tetramethylchroman-2-carboxylic acid) were purchased from Sigma-Aldrich Ltd., Poznan, Poland. Distilled water was used for meal preparation. For carbohydrate analysis, ready to use analytical kits such as β-Glucan Assay Kit (Mixed Linkage) and Total Starch Assay Kit (AA/AMG) of Megazyme International Ltd. (Wicklow, Ireland) were used for polysaccharides content assessment and purchased from Noack Polen Ltd., Warszawa, Poland. For free glucose determination in intact products, the GOPOD reagent (Megazyme International Ltd., Ireland) and glucose standard (Sigma Aldrich, Poznan, Poland) were used.

### 2.2. Functional Characteristic Measurement

#### 2.2.1. Granulometric Analysis

The size distribution was measured by granulometric analysis with vibratory sieve shaker Analysette 3 Spartan (Fritsch, Germany) on analytical sieves of 200, 500, 1000 and 2000 µm (200 mm diameter).

#### 2.2.2. Water Holding Capacity

The water holding capacity (WHC) and solubility index (WSI) determinations were based on AACC 88-04 method with some modifications for oat flakes and bran. Briefly, 30 mL of distilled water was poured into pre-weight 50 mL centrifuge tube and 5 g of sample was added. The sample was mixed, and then left for 24 h and centrifuged for 15 min at 3000 g. The solids residue was weighed and water capacity expressed as %/g of sample dry basis (d.b.).

#### 2.2.3. Water Solubility Index

The supernatant from centrifugation of WHC sample was carefully discarded into pre-weighed aluminum plates, weighed and put into moisture analyzer (MA30, Sartorius AG, Göttingen, Germany). The solid content in supernatant was calculated and expressed as water solubility index (WSI) of %/g of sample d.b.

### 2.3. Oatmeal Preparation

The sample preparation was described elsewhere [25]. Briefly, equal samples (50 g) of both types of raw materials (calculated per d.b.) were mixed with distilled water in 1:10 solid:liquid ratio and processed with microwaves of 750 W maximum power, 2.45 GHz frequency for 3 and 5 min with periodic stirring (values chosen in previous study [25]). As control, samples cooked in covered glass pot on heating plate until boiling, also with stirring, were taken. The stirring was provided at every heating minute for 10 s. Samples were centrifuged (10 min at 5000 g (MPW352R, MPW, Warszawa, Poland)) and the supernatant was used for the same day analysis.

### 2.4. Chemical Content Analysis

The chemical composition measurements were done in accordance with the AOAC (1996) for moisture, total ash, fat, and protein, while total carbohydrates were calculated by resting. The insoluble (IDF) and soluble (SDF) dietary fiber content was determined with enzymatic gravimetric method, based on AOAC 991.42, AOAC 993.19 and AACC 32-21 (AOAC, 1990). The total dietary fiber (TDF) content was calculated as a sum of SDF and IDF fractions. The β-glucan content in oat flakes and bran without processing as well as in supernatant after processing was measured according to procedure described in β-Glucan (Mixed Linkage) Assay Kit (Megazyme International Ireland, Ltd., Ireland). The starch content in the same samples was measured with Total Starch Assay Kit (Megazyme International Ireland, Ltd., Wicklow, Ireland). The moisture content of samples was determined gravimetrically (MA30, Sartorius AG, Germany). The available glucose was measure with GOPOD reagent (Megazyme International Ireland, Ltd., Ireland). Measurements were performed in triplicate.

### 2.5. Antioxidant Capacity Analysis

The antioxidant capacity of the water phase of meal (supernatant) was evaluated by three different assays based on electron transfer (ET) mechanism from freshly prepared sample without further dilution. The Folin–Ciocalteu reagent spectrophotometric method (765 nm) was used for the total phenolic-like (TPC-L) antioxidant capacity determination and expressed as gallic acid equivalents per gram of sample [26]. TEAC (Trolox equivalent antioxidant capacity) was measured with 2,2-diphenyl-1-picrylhydrazyl (715 nm) and 2,2′-azino-bis(3-ethylbenzothiazoline-6-sulfonic acid) (420) method and results were expressed as Trolox equivalent per gram of sample [26].

### 2.6. Glycemic Index Measurement

The assay was conducted using recognized GI method [27]. All subjects gave their informed consent for inclusion before they participated in the study. Twelve healthy, non-smoking (self-declared) participants aged 20–30 years took part in several experimental sessions. During each session, the participants were randomly assigned to consume 25 g of glucose (Sigma Aldrich, Poland) as reference and portion of 25 g available carbohydrate test meal. Glucose consumption was repeated three times by each participant to reduce the variation of mean GI values due to different glucose tolerance. The tested meal GI was evaluated in triplicate. After the fasting period, blood glucose readings were taken, the reference food and test foods were given and the participants were allowed to eat at a comfortable place within 10 min. The 3-day gap was maintained as the washout period to minimize any carryover effect and all participants were given written informed consent. Fasting blood glucose self-measurements were taken before the test foods were given to the participants (0 min), and further blood self-measurements were again collected at 15, 30, 45, 60, 90, 120 and 180 min after consuming the test foods. Each participant used individual disposal lancing device delivering capillary blood sample directly into strip which inserted into glucometer Accu Chek Active Blood Glucose Meter (Accu Chek, Indianapolis, IN, USA). Each measurement was taken in duplicate.

GI calculation of the incremental area under curve (iAUC) was done using Microsoft Excel (Version 2007, Redmond, WA, USA) with the trapezoid rule. All areas below the baseline were omitted. The GI, defined as the iAUC for each sample, was expressed as a percentage of the mean iAUC for glucose consumed by the same participant as his own reference. The resulting values for all participants were averaged to calculate the GI for each sample due to following equation:

$$\mathrm{GI}=\frac{\mathrm{area}\;\mathrm{under}\;\mathrm{the}\;\mathrm{blood}\;\mathrm{glucose}\;\mathrm{curve}\;\mathrm{of}\;\mathrm{tested}\;\mathrm{meal}}{\mathrm{area}\;\mathrm{under}\;\mathrm{the}\;\mathrm{blood}\;\mathrm{glucose}\;\mathrm{curve}\;\mathrm{of}\;\mathrm{reference}\;\mathrm{glucose}\;\mathrm{load}}\times 100$$

### 2.7. Statistical Analysis

The results were taken in triplicate and reported as means with standard deviation except for the blood glucose readings which were taken in duplicate. The analysis of variance (ANOVA) for mean values of nutritional components between types of raw materials was assessed with *p* < 0.05 significance level. Multivariate analysis (MANOVA) was performed to assess second order relation between sample and treatment for carbohydrate content, antioxidant activity, and glycemic index. The multivariate correlation coefficients were calculated between carbohydrate meal components and solids vs. antioxidant activity and total phenolic—similar to the antioxidant capacity of meals—with Statistica 12 (Statistica, Tulsa, OK, USA) at a probability level of *p* < 0.05.

## 3. Results

### 3.1. Characteristic of Nutritional Components

The nutritional analysis of oat bran and flakes from whole grain is presented in Table 1. The content of main components was similar to previous reports [28,29], except the carbohydrates, which were lower. As these compounds depend on, among others, the cultivation conditions, the comparison was made with product made from Poland cultivated oat. Both sample type nutrients contents were comparable to those previously reported [30,31].

### 3.2. The Product Functional Characteristic

**Figure 1:**
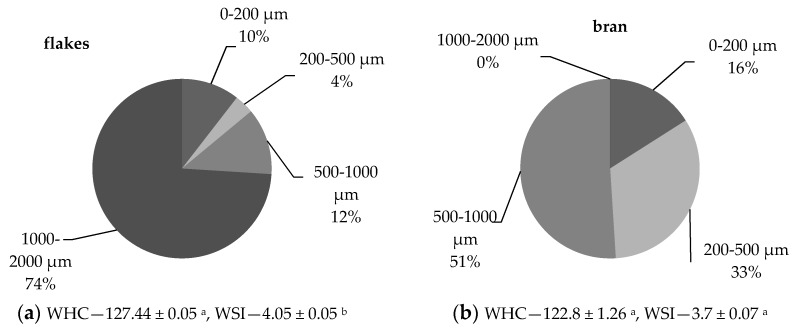
The particle size distribution of fractions (%) and water uptake parameters (%/g d.b.). (**a**) Flakes; (**b)** bran. WHC, water holding capacity; WSI, water solubility index. Mean values with different lowercase letters imply significant differences between means for the same parameter for different oat type products at *p* < 0.05.

The two studied oat breakfast products came from completely different processing streams, which means oat flakes were from whole grain processing, while bran was from flour production by grain milling. Therefore, their morphology and morphology-resulting functional characteristics were different, which probably could impact further postprandial glycemia. The size distribution of particles and water interaction values are presented in Figure 1.

The distribution analysis revealed that fraction above 1000 µm was abundant in flakes and 1.5 times higher than in bran. The lowest size fraction—below 200 µm—was higher in bran than in flakes. The oat grains for flakes manufacturing are firstly gelatinized during steaming, then, as elastic, are flattened. Finally, they are sieved to remove smaller particles, therefore, such fine fraction is probably a result of a crush during storage and handling in a package. Bran, being peeled off from the previously dehulled grain, contains a high level of starch, which is fixed to aleuronic layer due to the high fat content of oat. However, opposite to the flakes, the fine endosperm fraction from native untreated grain may deliver much lower postprandial glycemia increase.

### 3.3. The Carbohydrate Release

**Figure 2:**
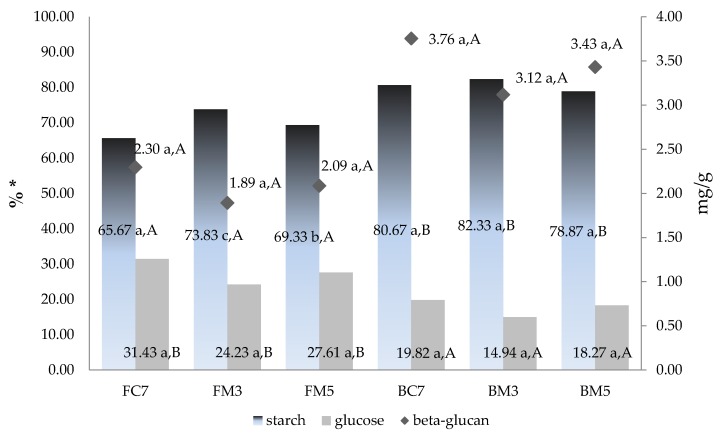
Effect of microwave irradiation on carbohydrate release during meal heating. FC7 and BC7, flakes and bran convection heated for 7 min; FM3, FM5, BM3, and BM5, flakes and bran microwave heated for 3 or 5 min; * per the dry basis of solids weight. The different lowercase letters indicate statistically significant difference between treatment types within the same sample type; different upper-case letters indicate statistically significant difference between sample types within the same treatment.

The changes in starch, glucose and β-glucan content (Figure 2) were assessed in relation to treatment and sample type. Depending on heating method, the starch content in a liquid phase of the meal was altered, being the highest in a sample irradiated for 3 min. The lowest values were obtained for 5 min heated flakes samples, both microwave and conventionally, while there was no statistically significant difference between starch release from bran, which had higher starch content than flakes.

The glucose release was dependent on neither treatment type nor time—the only difference was the result of sample type. The total content of glucose was about 1.5 times higher in flakes which can result from the previous gelatinization of starch and easier hydrolysis to glucose within treatment conditions. The highest release of β-glucan into solution was also observed in bran, however the content was independent from applied conditions.

In all samples, strong and negative (−0.77) statistically significant correlation was observed between starch and glucose in flakes meal, which was confirmed by partial coefficient value (−0.89) after considering other interactions among compounds. In addition, between glucose and β-glucan, there was strong positive correlation, indicating combined release of both in flakes. In bran, the β-glucan release was not correlated with other carbohydrate transport (Table 2).

### 3.4. Antioxidant Capacity

**Figure 3:**
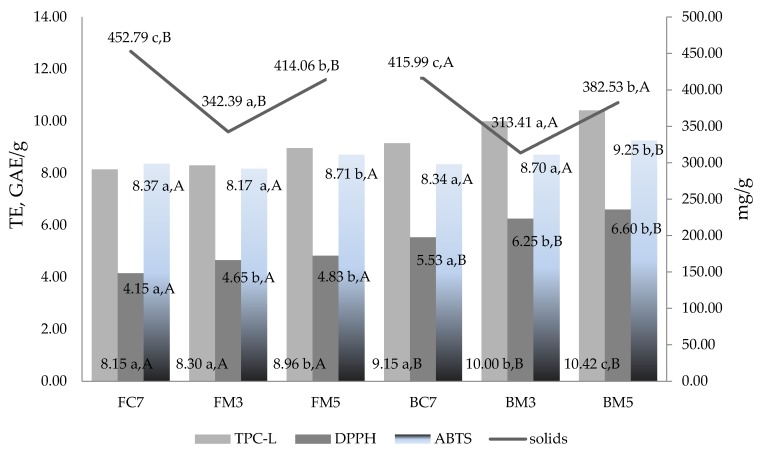
Effect of microwave irradiation on antioxidant capacity of solids released during meal heating. FC7 and BC7, flakes and bran convection heated for 7 min; FM3, FM5, BM3, and BM5, flakes and bran microwave heated for 3 or 5 min. The different lowercase letters indicate statistically significant difference between treatment types within the same sample type; different uppercase letters indicate statistically significant difference between sample types within the same treatment.

The antioxidant capacity of solids released into solution was assessed in relation to sample type, treatment, and temperature impact. The TPC-L, DPPH and ABTS values showed important differences due to either the heating method or product type (Figure 3).

The highest content of solids in the supernatant was observed for flakes conventionally cooked (7 min) while the lowest one was for 3 min microwave heated bran. The lowest values were obtained for microwave heated samples for both products types, which suggests that microwave heating with shorter processing time does not permit such extensive release of solids into the solution. This was comparable to results obtained by Yiu et al. [32]. In addition, bran vs. flakes reveals the superiority of antioxidant activity with lower solids content which suggests higher small antioxidant molecules extraction. As the assays were made on freshly made meal, such antioxidant activity can reflect the total antioxidant potential easily available while eating the meal.

The partial coefficients of multivariate correlation analysis (Table 3) revealed the strong negative correlation between solids and DPPH measured activity (−0.72) in flakes meal as well as strong correlation (0.9 and 0.78) between DPPH and ABTS vs. TPC-L values. For bran, only direct positive and strong correlation (0.78) with ABTS was observed. The analysis of partial coefficients revealed a further indirect strong correlation between either TPC-L and DPPH or TPC-L and ABTS in bran. The partial correlations coefficient measures the strength of the linear relationship between the variables being first adjusted for their relationship to other variables.

### 3.5. Glycemic Index

**Figure 4:**
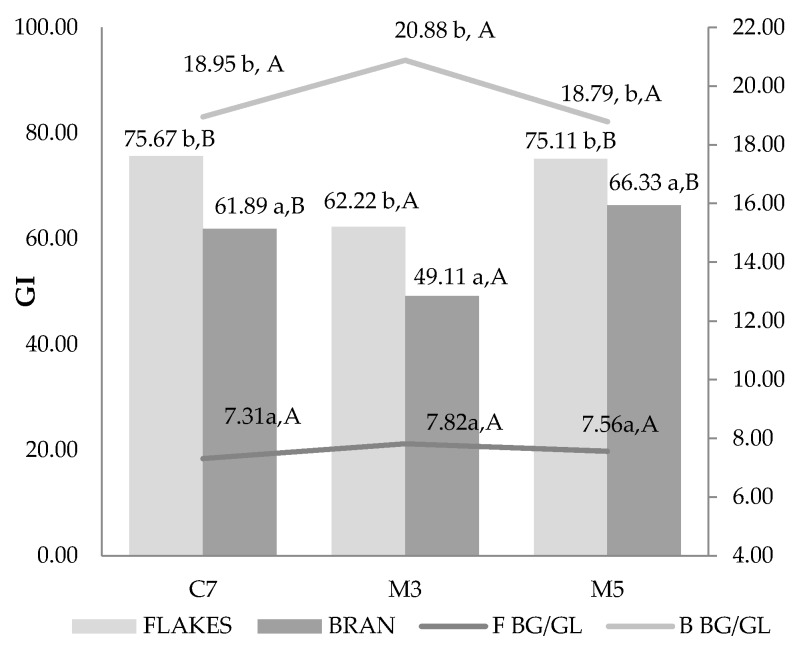
Glycemic index values. C7, convection heated for 7 min; M3 and M5, microwave irradiated for 3 or 5 min; BG/GL, β-glucan/glucose ratio in liquid part of meal. The different lowercase letters indicate statistically significant difference between treatment types within the same sample type; different uppercase letters indicate statistically significant difference between sample types within the same treatment.

The measurement of glycemic index change during 180 min after each meals consumption gives additional insight to bioaccessibility of nutrient from meal prepared with different heating methods (Figure 4).

The glycemic index analysis has reveals that either heating method or sample type used have impacted significantly on a postprandial glucose response. The unexpected results were obtained for microwave irradiated samples, where there was no statistically significant difference between convectional heating method and longer microwave irradiation. It may suggest that both types deliver a substantial amount of easily digested carbohydrates. The difference was noted for shorter microwave heating in both sample types flakes and bran. The bran porridge from each treatment has resulted in lower postprandial glucose increase, being the lowest for 3 min microwave heated samples. The β-glucan/glucose ratio was about 2.5–3 times lower in all bran samples, indicating the important contribution of β-glucan viscosity.

## 4. Discussion

Usually, nutritional information provided by manufacturers in form of so-called “nutritional facts” focuses on delivering the basic knowledge of food ingredients as protein, fat or carbohydrates. However, following the rising knowledge of several particular carbohydrate components’ impacts on health, the nutrition facts also become more informative, therefore such fractions as dietary fiber (both soluble and insoluble) or β-glucan content also appear. Nowadays, consumers are well aware that oat flakes are much more nutritious than any other typical breakfast cereal, which are frequently formed from extruded flour with very high glycemic index. However, oat bran, as rich in β-glucan and fiber, is also gaining consumer attention and is starting to be considered a breakfast product.

Comparison of two types of oat product based on carbohydrate ratios helps in assessing their potential behavior during cooking. The three ratios—carbohydrates/TDF, starch/β-glucan, and glucose/β-glucan—were lower for bran than flakes, which can cause the much lower postprandial glycemia increase. The only value of ratio which was higher for bran than for flakes was starch/glucose ratio, which, due to lower glucose content, was finally higher in bran. The glucose initial content difference may result from previous (industrial level) grain treatment. Flakes are produced from grains previously steamed and then rolled while still warm [33]. Such pretreatment leads to partial gelatinization of starch which may justify the higher level of initial glucose content as result of starch hydrolysis. It may also suggest much higher glycemic index due to the existence of pregelatinized starch which is easily available to digest. The heating and stirring during meal preparation lead to the transfer of dissolved components into solution. The resulting liquid reflects the part of meal, which is more easily accessible by enzymes and more available to digestion than the solid part, which, in the case of oat porridge, is additionally glued together due to high viscosity of particles. Therefore, the liquid part can be supposed to be mainly responsible for initial postprandial glycemia. Mackie et al. [18], comparing two types of oat porridge, observed that oat flake porridge showed significant signs of sedimentation in stomach immediately post consumption with lacking initial fluid phase which was probably due to faster absorption of the fluid phase. Due to its high molecular weight, β-glucan needs a lot of available water to hydrate the chains; therefore, competition with alfa-glucan (starch) with same requirements but being predominant in flakes may hinder its release into solution. Moreover, the physical form of whole grain flakes being only slightly ruptured during rolling may restrict the easy transfer of β-glucan to the solution. Mackie et al. [18] observed that the increased viscosity in the stomach was not created by the starch or β-glucan but rather by the intact structure of the flakes [34].

Data from water extracts of oat products assessed for antioxidant capacity are scarce, probably because research mainly focuses on the total antioxidant potential of raw material and use one of the organic solvents to extract all the possible active compounds. However, during eating, the probability of achieving the same level of antioxidant activity is very low. The different solvent used for meal preparation, i.e., water vs. organic solvent, the real morphological form of food and its fragmentation level impact food accessibility for the human digestive system. Therefore, the assessment of its soluble fraction profile may lead to quick evaluation of the most accessible part of the food. Zieliński and Kozłowska [35] found that for whole grain of oats the number of solids extracted by water was higher as comparing to extracted by 80% methanol, however the 80% methanol extract had higher TPC (17.6 vs. 1.5 µg catechin/mg lyophilizate, respectively) and exhibited higher antioxidant capacity (0.08 vs. 0.03 µmol Trolox/lyophilizate, respectively).

Stevenson et al. [36], when assessing the effectiveness of used solvents for the antioxidants extraction from enriched oat β-glucan fiber (ground oat bran concentrate), concluded that obtained TPC of defatted oat bran water extracts was 9.2 mg/g, being 10 times higher than methanolic and acetone extracts obtained by other research [28,29]. The DPPH value of the same extracts was the highest for 50/50 *w*/*w* extracts and the statistically significant closest to this result were both water extracts—from defatted and non-defatted OBC. As water itself reacts neither with Folin–Ciocalteu reagent nor with DPPH and ABTS compounds (oppositely to acetone and methanol), it is supposed that antioxidant activity of water extracts is strongly correlated with the solids content of oat meals liquid phase. The ANOVA analysis of functional characteristic (WSI and WHC) of both products also revealed the significant differences in solids release even in mild temperature conditions of the assay (room temperature).

The functional properties of oatmeal ingredients significantly influenced the glycemic response [34]. The oat flake meals were probably digested in two stages—first by absorption of the solution and then after emptying the stomach by further digestion in intestines, as such behavior in the stomach was confirmed by other researchers [37]. Microwave heating resulted in similar values as conventional heating, which suggests that long-time treatment above gelatinization temperature of oat starch delivers easily digested and absorbed carbohydrates. The GI lowest values for meal heated for short time in microwaves may be related either with short time processing with less extensive stirring and lower particle crushing or shorter exposure to gelatinization temperature in which starch granules swells releasing amylose chains which are further subjected to α-amylase hydrolysis. The obtained results are in agreement with Mackie et al. [18] who observed that, at 35 min after ingestion, the flake porridge was associated with lower hunger, whereas at 180 min the flake porridge got the highest ratings of hunger and desire to eat.

The oat bran meal revealed expectedly lower values, however the samples MW heated for 5 min gave the very same response and even higher than longer conventionally cooked bran. The higher glycemic response is probably connected with particle size level and easier starch accessibility in small and open (comparing to morphological form of flakes) samples of bran. Therefore, increased starch hydrolysis in the intestine may finally lead to a similarity in general response. However, also due to morphological form of bran, the availability of starch to enzymatic hydrolysis is continuous contrary to flakes which morphological form restricts full enzyme access. The lower GI values for short time microwave heated meal also confirms this suggestion because a part of the flour in bran was not excessively gelatinized and made accessible comparing to 5 min one. The other aspect that may influence the glucose appearance and transport is β-glucan content, which in ratio to free glucose content in a meal was much higher for bran than flakes (BG/GL Figure 4—calculated as liquid phase β-glucan content/glucose). It can be supposed that in the digestive system this ratio can be somehow repeated, which can mainly be due to morphological restriction in flake particles. Although bran particles are small and easily available for digestive enzymes, they are the same way available for β-glucan extraction by fluids in the gastrointestinal track. Therefore, for postprandial glycemic response, the mechanistic activity of β-glucan, its viscosity as well as metabolic interaction with Peyer’s patches in intestinal layer should also be taken into account [38].

## 5. Conclusions

The β-glucan release in the conditions of the experiment was independent of applied factors (sample type and treatment). However, the significant difference was observed for starch/glucan ratio. The lowest starch content was obtained for conventionally heated oat flakes, which means that in such food was the highest amount of easily available carbohydrates. The 5 min microwave heated flakes also had high easily available carbohydrate load, although lower than conventionally cooked. The 3 min microwave heating of flake and oat meals revealed the highest content of starch and the lowest GI, being the meal most equilibrated in carbohydrates. Bran vs. flakes revealed the much higher content of starch and generally much lower GI during all preparation methods. The antioxidant activity was the highest for bran samples heated with microwaves for 5 min. For each type of product an adequate processing method should be used, as they impact differently the carbohydrate availability and resulting glycemic index. Moreover, the heating method can promote or hinder carbohydrate release in the digestive tract. Obtained results suggest that properly applied microwave heating can provide substantial support for nutritionally valuable meal preparation.

## Figures and Tables

**Table 1:** Nutrients content of used raw materials and carbohydrates ratio.

Nutrient *	F		B	
protein	13.88 ± 0.71 ^a^	TCH/TDF 2.77	16.77 ± 2.32 ^a^	TCH/TDF 2.01
fat	9 ± 0.98 ^a^		7.14 ± 1.57 ^a^	
TCH	48.48 ± 1.74 ^b^	ST/GL 46.19	42.68 ± 1.13 ^a^	ST/GL 64.67
starch	47.03 ± 1.69 ^b^		41.39 ± 1.09 ^a^	
glucose	1 ± 0.04 ^b^	ST/BG 9.98	0.6 ± 0.02 ^a^	ST/BG 6.64
TDF	17.6 ± 1.21 ^a^		21.3 ± 1.2 ^b^	
β-glucan	4.73 ± 0.38 ^a^	GL/BG 0.22	6.1 ± 0.48 ^b^	GL/BG 0.1
ash	1.64 ± 0.08 ^a^		2.08 ± 0.21 ^b^	
energy load ^#^	312.48 ± 7.94 ^b^		287.75 ± 3.17 ^a^	

F, flakes; B, bran; TDF, total dietary fiber; TCH, total carbohydrates; ST, starch; GL, glucose; BG, β-glucan; TCH/TDF, ST/GL, ST/BG, and GL/BG, mutual ratios of relevant carbohydrates; mean values with different lowercase letters imply significant differences between means in row at *p* < 0.05. ^#^ calculated, * per the dry basis of solids weight.

**Table 2:** Pearson correlation coefficients and partial correlation coefficients between carbohydrates in meal.

		ST	GL	BG
ST	F	-	−0.77 *	−0.37_ns_
	B	-	−0.06_ns_	−0.32_ns_
GL	F	*−0.89* **	-	0.82 **
	B	-	-	−0.57_ns_
BG	F	*0.74* *	*0.91* **	-
	B	-	-	-

F, flakes; B, bran; ST, starch; GL, glucose; BG, β-glucan; values in italics represent partial coefficients, _ns_: *p* > 0.05; *: *p* < 0.05; **: *p* <0.01.

**Table 3:** Pearson correlation coefficients and partial correlation coefficients between solids and their antioxidant capacity.

		Solids	TPC-L	DPPH	ABTS
solids	F	-	0.64_ns_	−0.76 *	−0.35_ns_
	B	-	−0.49_ns_	−0.46_ns_	−0.17_ns_
TPC-L	F	-	-	0.90 **	0.78 *
	B	-	-	0.85_ns_	0.88 **
DPPH	F	*0.73* *	*0.73* *	-	−0.60_ns_
	B	-	*0.80* *	-	0.62_ns_
ABTS	F	-	-	-	-
	B	-	*0.92* **	-	-

F, flakes; B, bran; values in italics represent partial coefficients, _ns_: *p* > 0.05; *: *p* < 0.05; **: *p* < 0.01.
